# The effect of *Toxoplasma gondii* infection in parental male mice on the transcriptome of their offspring’s brain

**DOI:** 10.1186/s13071-026-07302-7

**Published:** 2026-02-26

**Authors:** Ya-Nan Li, Hang Sun, Jian Ma, Xin-Yuan Zhou, Xiao-Man Xie, Huan-Huan Xie, Yan-Han Hou, Hong-Jie Dong, Gui-Hua Zhao, Chao Xu, Hong-Tao Li, Kun Yin

**Affiliations:** 1 Shandong Institute of Parasitic Diseases, Shandong First Medical University and Shandong Academy of Medical Sciences, Jining, 272033 Shandong China; 2 School of Public Health, Shandong First Medical University and Shandong Academy of Medical Sciences, Jinan, 271016 Shandong China; 3 Joint Laboratory of Shandong Institute of Parasitic Diseases and Jining City Public Health Medical Center, Jining, 272033 Shandong China; 4 Institute of Brain Science and Brain-Inspired Research, Shandong First Medical University and Shandong Academy of Medical Sciences, Jinan, 271016 Shandong China

**Keywords:** *Toxoplasma gondii*, Brain tissue, Transcriptome, Intergenerational effects

## Abstract

**Background:**

To investigate the mechanisms and intergenerational effects of *Toxoplasma gondii* infection in parental male mice on the transcriptome of the brain of their offspring.

**Methods:**

Male parental mice were infected with the *T. gondii* strain *TgCtwh6* and then mated with healthy female mice to produce offspring F1. Three independent and comparable groups were established: infected male mice (M) versus F1 male generation (F1♂) (M vs. F1♂), healthy female mice (F) versus F1 female generation (F1♀) (F vs. F1♀), and parental generation (P) versus F1 generation (F1) (P vs. F1). RNA was extracted from the brain tissues of both parental and offspring mice for transcriptome sequencing, screening for differentially expressed genes (DEGs) common to all three groups. DEGs were identified and validated by quantitative reverse transcription polymerase chain reaction (qRT-PCR). Furthermore, functional analyses including Gene Ontology (GO), Kyoto Encyclopedia of Genes and Genomes (KEGG), and evolutionary genealogy of genes: Nonsupervised Orthologous Groups (eggNOG) classification were performed to reveal the potential functions of DEGs in mice and genes in biological processes, key metabolic or signaling pathways, which provide a molecular basis for further studies on how to affect transcriptional expression in offspring.

**Results:**

An overlap in gene expression was observed among the M versus F1♂, F versus F1♀, and P versus F1 comparisons. Collectively, these three comparisons identified 66 DEGs that were consistently altered across all groups, comprising 19 upregulated and 47 downregulated genes. GO analysis revealed that these DEGs were predominantly enriched in categories such as identical protein binding, positive regulation of NF-kappa B transcription factor activity, and membrane raft. KEGG analysis further indicated that the majority of enriched pathways were associated with immune responses, including those involved in viral infection pathways. qRT-PCR was employed to validate the expression changes of key genes.

**Conclusions:**

*T. gondii* infection of male parental mice significantly downregulates gene expression in the brain tissue of their offspring and negatively regulates the immune system and signal transduction pathways. This study provides valuable insights into the intergenerational effects of *T. gondii* infection and highlights the importance of further research in this critical area.

**Graphical Abstract:**

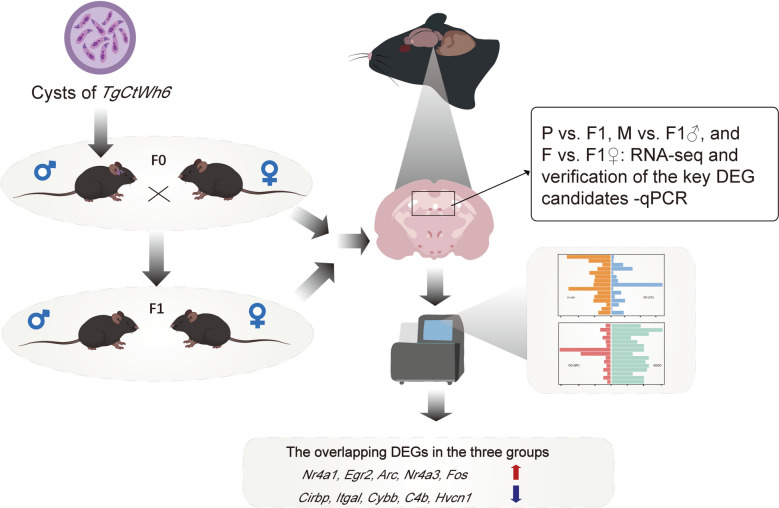

**Supplementary Information:**

The online version contains supplementary material available at 10.1186/s13071-026-07302-7.

## Background

*Toxoplasma gondii* is a globally distributed, foodborne, opportunistic intracellular parasite that infects a wide range of warm-blooded hosts, including humans. It undergoes sexual reproduction in the intestinal epithelial cells of cats and excretes oocysts, which can be transmitted to other hosts through contaminated water or food [1, 2]. Human infection primarily occurs through the consumption of undercooked meat from infected animals or exposure to food and water contaminated with cat feces [3, 4]. Statistically, about 30% of the global population is infected with *T. gondii* [5], and the seroprevalence rate in the Chinese population is about 0.72–23.41% [6].

*T. gondii* infections are categorized into two primary forms: acute and chronic. During acute infection, tachyzoites rapidly invade vital organs such as the brain, lungs, and eyes, potentially leading to severe encephalitis or even mortality in immunocompromised individuals [7]. In contrast, chronic infection is characterized by the transformation of tachyzoites into bradyzoites, which form cysts primarily residing in the brain and muscle tissues of the host [8]. Research has demonstrated that *T. gondii* cyst distribution in the brain predominantly affects the frontal lobe and hippocampus [9], potentially increasing the risk of psychiatric disorders, such as depression, anxiety, and schizophrenia, by altering central nervous system (CNS) function and modifying host behavior [10]. *T. gondii* strains are generally classified into types I, II, and III [11], with type II being more prevalent among populations in Europe and North America [12]. African populations, however, are more commonly affected by atypical clonal strains of *T. gondii* [13]. In China, Chinese type I is the predominant strain, encompassing both highly virulent (*TgCtwh3*) and less virulent (*TgCtwh6*) variants [14]. In the present study, the *TgCtwh6* strain was utilized to establish an experimental mouse model aimed at investigating the behavioral effects of *T. gondii* infection and its underlying mechanisms.

In recent years, evidences have emerged indicating that *T. gondii* infection not only affects the individual host but may also exert long-term consequences on subsequent generations [15]. For instance, *T. gondii* infection can result in a range of long-term epigenetic modifications in host cells [16]. Specifically, the levels of small RNA molecules carried in the sperm of male mice infected with *T. gondii* may be altered [17], and such modifications have the potential to affect brain development and behavioral phenotypes in offspring [18]. Further, more concerning is the observation that epigenetic changes in initially infected males may persist into the third generation, suggesting that *T. gondii* infection may exert transgenerational effects on offspring through epigenetic information transmitted via sperm. Notably, it has been reported that behavioral changes in male rodent hosts caused by chronic *T. gondii* infection, such as anxiety-like phenotype, depression-like behavior, and impairments in spatial working memory, can also be observed in both the F1 and F2 generation of mice [17]. However, owing to the significant fertility impairment observed in male animal models following infection, there have been no reports to date on brain transcriptome changes in the F1 generation derived from infected males. As a result, whether infection-induced transcriptional alterations in the offspring’s brain are associated with corresponding behavioral and cognitive phenotypes remains unclear.

Thus, in this study, we aim to investigate the molecular basis of *T. gondii*-induced behavioral disorders in offspring through brain transcriptomic analysis to compare gene expression profiles between the F1 generation and their parents. Infected males were mated with uninfected healthy females, and the required number of F1 offspring was successfully obtained. Brain transcriptome analyses were performed on adult F1 offspring, and their gene expression profiles were compared with those of the parental generation. The expression levels of selected genes were further validated, providing novel insights into the intergenerational consequences of *T. gondii* infection.

## Methods

### Laboratory animals

Female *C57BL/6* mice were utilized as breeding subjects, and healthy male *C57BL/6* mice weighing 20–25 g at 8 weeks of age were chosen as infection subjects. All mice were obtained from Jinan Pengyue Laboratory Animal Center [Production License No. SCXK (Lu) 20220006] and housed in specific pathogen-free (SPF)-grade animal facilities under controlled conditions, including a 12-h light/dark cycle, ambient temperature of 22 ± 2°C, relative humidity of 50%–60%, and *ad libitum* access to food and water. Prior to the initiation of the experiment, all mice were acclimatized to the laboratory environment for 1 week. The experimental protocol was approved by the Institutional Animal Care and Use Committee (IACUC) of the unit (Approval No. W202402270064).

### *T. gondii* strains and infections

The weakly virulent *TgCtwh6* strain of *T. gondii* was maintained by our laboratory. Male mice were intraperitoneally inoculated with *TgCtwh6* tachyzoites following this procedure: frozen tachyzoites were thawed and inoculated into Vero cells for propagation. Tachyzoites released through cell lysis were subsequently harvested. The infection dose was set at 1 × 10^3^ tachyzoites per mouse (0.1 mL, intraperitoneal injection), with the tachyzoite concentration adjusted to 1 × 10^5^ tachyzoites/mL using phosphate-buffered saline (PBS). Control animals received an equivalent volume of sterile PBS.

### Animal grouping and breeding

At 14 days after *T. gondii* infection, infected male mice were mated with uninfected female mice at a ratio of 1:3 in shared cages. The date of cohabitation and the birth dates of the offspring were documented. We paired five litters in parallel; however, a total of 11 F1 generation mice were obtained from only one litter, comprising six males and five females, all exhibiting a 100% survival rate and healthy condition. Following weaning (at 21 days postpartum), the F1 generation mice were housed individually for subsequent behavioral and molecular biological analyses. All of the F1 offspring from a single successful litter were subjected to behavioral analysis. Following this, a subset of these offspring (*n* = 3 males and *n* = 3 females) was randomly selected, and paired with parental samples to form three independent comparison groups (M versus F1♂, F versus F1♀, P versus F1) for transcriptome sequencing. RNA was extracted individually from the brain of each of the six animals, without pooling tissues, ensuring that each sequenced sample represents an independent biological replicate.

### Transcriptomic analysis

Brain Tissue Sampling: the mice were executed via cervical dislocation, and brain tissues (specifically the frontal lobe and hippocampus) were rapidly collected. Brain tissue samples were immediately frozen in liquid nitrogen and stored at −80 °C until further processing.

RNA extraction and RNA-sequencing: total RNA was extracted from brain tissue using TRIzol reagent (Invitrogen, USA) according to the manufacturer’s instructions. The concentration and purity of the RNA were assessed using a NanoDrop 2000 spectrophotometer. RNA integrity was further evaluated using an Agilent 2100 Bioanalyzer. The RNA samples with an RNA Integrity Number (RIN) value less than 7.0 were excluded from further sequencing. Sequencing libraries were prepared using the KAPA RNA Hyperprep kit with RiboErase (HMR) and sequenced on an Illumina NovaSeq 6000 as 150 bp pair-end (PE) reads. About 40 million reads were generated for each sample.

RNA-sequencing data analysis: the reads from total RNA-seq libraries were trimmed for adapter sequences and aligned to the GRCm38/mm10 genome using HISAT2 (v2.2.1) with proper capture of RNA strand orientation. Then we processed the aligned reads with StringTie76 to quantify the gene expression using GENCODE M23 annotation. Gene count files were generated using the StringTie prepDE.py script, and then further processed with DESeq2 to identify differentially expressed genes (counts > 1, Fold Change > 1.5, adj. *P* value < 0.05).

### Functional enrichment analysis

Based on gene transcriptome sequencing data, differential expression analysis was conducted between parental and offspring samples using the DESeq2 tool to identify genes exhibiting significant DEGs. The screening criteria included a Fold Change > 1.5 and a Benjamini–Hochberg (BH)-corrected *P*-value (*Q*-value) < 0.05. The DEGs identified through this screening were subsequently subjected to functional annotation and enrichment analyses, including GO enrichment analysis, KEGG pathway analysis, and homology-based protein cluster annotation (eggNOG).

GO enrichment analysis: this classification system categorizes DEGs into three functional domains—Biological process (BP), Cellular component (CC), and Molecular function (MF)—to elucidate their specific roles within the cellular context, such as catalytic activity, binding capacity, and biological functions related to signal transduction or cell division.

KEGG pathway analysis: through enrichment analysis of metabolic pathways, signaling pathways, and disease-associated pathways, this approach enables the investigation of synergistic regulatory interactions among DEGs within biological systems. Such analysis provides insights into potential gene functions during disease progression or drug action mechanisms.

EggNOG enrichment analysis: as an extension of the COG/KOG database, eggNOG facilitates comprehensive functional characterization of DEGs. It supports more complete gene function annotation and evolutionary analysis by incorporating orthologous groups across eukaryotic, prokaryotic, and viral Orthologous Groups (OG), thereby enabling functional exploration from an evolutionary perspective.

### cDNA synthesis

A total of 1 μg of RNA was reverse transcribed into cDNA using the PrimeScript™ RT Kit (Takara, Japan). The reaction mixture, with a total volume of 20 μL, contained 4 μL of 5 × PrimeScript Buffer, 1 μL of RT Primer Mix, 1 μL of PrimeScript RT Enzyme Mix, and 14 μL of RNA template. The thermal cycling conditions were as follows: 15 min at 37 °C, followed by 5 s at 85 °C.

### Quantitative reverse transcription polymerase chain reaction

The relative expression levels of the target genes were quantified using the SYBR Green method, with the glyceraldehyde-3-phosphate dehydrogenase (*GAPDH*) gene as an internal reference gene. Primer sequences were designed using Primer 5.0 software and presented in Table 1. The reaction mixture, with a total volume of 20 μL, consisted of 10 μL SYBR Green Mix, 1 μL cDNA template, 0.5 μL of forward and reverse primers (10 μM each), and 8 μL ddH_2_O. The thermal cycling conditions were as follows: initial predenaturation at 95 °C for 5 min, followed by 40 cycles of denaturation at 95 °C for 15 s, annealing at 60 °C for 30 s, and extension at 72 °C for 30 s. Relative gene expression levels were calculated using the 2^−ΔΔCt^ method.

**Table 1:** Primer sequence for qRT-PCR

Nr4a1	Forward	GCGGGCAGAGACGCGA
	Reverse	GCTTGAATACAGGGCATCTCCACT
Egr2	Forward	ATCACAGGCAGGAGAGACTGC
	Reverse	CCTCCCAGTTCACCATTGGG
Arc	Forward	GAACCTCAACTTCCGGGGAT
	Reverse	ACTGGTATGAATCACTGGGGG
Cirbp	Forward	CTACTATGCCAGCCGGAGTC
	Reverse	GCTCTGAGGACACAAGGGTT
Cybb	Forward	CCATCCATGGAGCTGAACGA
	Reverse	GCCAAAACCGAACCAACCTC
Itgal	Forward	ACTTCCACTTCCCGATCTGC
	Reverse	TCTCAGGATAGGCTGCATGG
C4b	Forward	GTGACAACAAGGGAGACCCC
	Reverse	GTCCTAAGGCCTCACACCTG
Hvcn1	Forward	GTGTGCCTGAGTCTCTACCC
	Reverse	CTGCGAGTGACGGCCTTT
GAPDH	Forward	ACCCTTAAGAGGGATGCTGC
	Reverse	CCCAATACGGCCAAATCCGT

### Statistical analysis

Statistical analysis was conducted using SPSS 22.0 software. A *P*-value less than 0.05 was considered statistically significant. Graphical representations were generated using GraphPad Prism 10.0 software.

## Results

### Acquisition and quality control of RNA from mouse brain tissue

Based on the behavioral disorder phenotypes observed in F1 mice [17], we dissected the hippocampus and frontal cortex of all mice—brain regions critically involved in learning, memory, and higher cognitive functions—for total RNA extraction and transcriptome sequencing. Quality assessment of the sequencing data confirmed that all samples met the required analytical standards: each sample generated an average of more than 21 million reads, with Q20 and Q30 base call accuracies exceeding 98.9% and 95.5%, respectively (Additional file 1: Supplementary Table S1), thereby ensuring the reliability and robustness of downstream transcriptomic analyses.

Following quality control filtering, the raw sequencing data from all samples yielded 732.33 Mb of high-quality clean reads. Each sample contained more than 41.36 Mb of clean reads, with over 41.31 Mb of reads aligning to the reference genome (mapped reads), resulting in a mapping rate ranging from 96.3% to 97.3%. Further analysis revealed that the number of uniquely mapped reads exceeded 37.51 Mb per sample, accounting for 90.3%–92.3% of the total reads, whereas the number of multi-mapped reads ranged from 2.15 to 2.65 Mb, representing 4.6%–6.3%. In addition, approximately 51.5% of the mapped reads were evenly distributed between the positive and negative strands of the reference genome (Additional file 2: Supplementary Table S2).

### Identification of DEGs among parental and offspring mouse groups

Independent pairwise comparisons were initially conducted, including “P versus F1”, “M versus F1♂”, and “F versus F1♀”. To enhance the reliability of the results, DEGs shared across all three comparison groups were selected for further analysis. In the M versus F1♂ group, the top ten DEGs were *Cxcl9*, *Gbp10*, *Jchain*, *Cd74*, *C3*, *H2-Aa*, *H2-Eb1*, *Iigp1*, *Gbp2*, and *Igtp*, all of which exhibited a downregulated expression pattern. In the F versus F1♀ group, the top ten DEGs were *Nr4a1* (Nuclear receptor subfamily 4 group a member 1), *Nr4a3* (Nuclear receptor subfamily 4 group a member3), *Fosl2*, *Egr2* (Early growth response protein 2), *Egr4*, *Gm28551*, *Sik1*, *Hspb1*, *Fosb*, and *Arc* (Activity-regulated cytoskeleton-associated protein), all of which showed upregulated expression. In the P versus F1 group, the top five most significantly upregulated genes were *Nr4a1*, *Egr2*, *Arc*, *Nr4a3*, and *Fos* (Fos proto-oncogene, AP-1 transcription factor subunit), whereas the top five most significantly downregulated genes were *Cirbp* (Cold inducible RNA binding protein), *C3*, *Cd74*, *H2*-*Eb1*, and *H2*-*Ab1*. Following a comparative analysis of the three groups, a total of 66 DEGs were identified in the overlapping intersection, comprising 19 upregulated and 47 downregulated genes. The results demonstrated that the number of downregulated genes exceeded that of upregulated genes, which is consistent with the overall trend observed across the three experimental groups, indicating a predominant suppression of gene expression. Among the overlapping genes identified in the three pairwise comparisons, the top five upregulated genes with the most significant expression changes were *Nr4a1*, *Egr2*, *Arc*, *Nr4a3*, and *Fos*, whereas the top five downregulated genes were *Cirbp*, *Itgal* (Lymphocyte function-associated antigen 1), *Cybb* (Cytochrome b-245 heavy chain), *C4b*, *Hvcn1* (Hhydrogen voltage gated channel 1) (Table 2), and *Gpr84* (G protein-coupled receptor 84) also exhibited significant downregulation.

**Table 2:** Group comparison of DEGs

Symbol	M versus F1♂		F versus F1♀		P versus F1		Regulated
	*P* value	log_2_FC	*P* value	log_2_FC	*P* value	log_2_FC	
*Nr4a1*	4.03 × 10^–21^	2.43	6.67 × 10^−24^	1.64	3.64 × 10^−20^	1.90	up
*Nr4a3*	7.61 × 10^–10^	1.90	5.08 × 10^− 21^	1.26	2.50 × 10^−9^	1.31	up
*Egr2*	5.66 × 10^–10^	2.61	3.28 × 10^−18^	2.60	1.55 × 10^−16^	2.63	up
*Fos*	2.26 × 10^–12^	2.68	9.78 × 10^−6^	2.02	1.17 × 10^−8^	1.57	up
*Arc*	3.29 × 10^–32^	1.77	9.09 × 10^−11^	1.31	9.17 × 10^−15^	2.17	up
*Cirbp*	5.58 × 10^–23^	−1.48	2.90 × 10^−12^	−9.09 × 10^−1^	3.71 × 10^−9^	−1.05	down
*Itgal*	6.03 × 10^–194^	−6.74	0.005565	− 8.30 × 10^−1^	3.01 × 10^−8^	−4.64	down
*Cybb*	8.91 × 10^–289^	−7.28	0.039156	− 7.11 × 10^−1^	5.22 × 10^−8^	−5.52	down
*C4b*	6.81 × 10^–239^	−4.64	1.68 × 10^−6^	−6.97 × 10^−1^	1.18 × 10^−7^	−3.89	down
*Hvcn1*	4.10 × 10^–87^	−5.75	0.000882	−9.00 × 10^−1^	1.28 × 10^−6^	−3.02	down

An intersection analysis was conducted on the DEGs identified in three pairwise comparisons M versus F1♂, F versus F1♀, and P versus F1, to identify consistently dysregulated across all three contrasts

### GO and KEGG enrichment analysis of DEGs

**Fig. 1:**
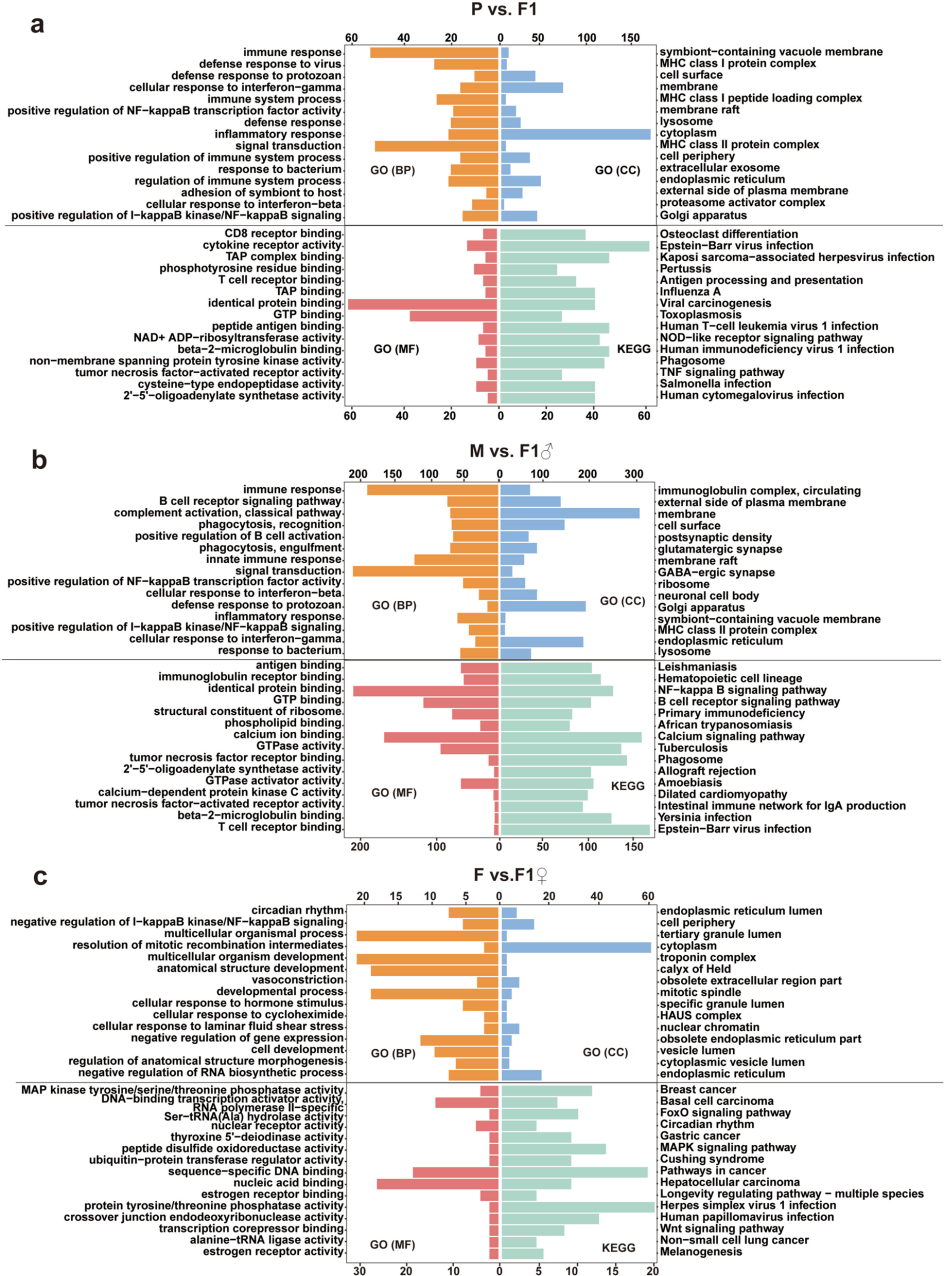
Figures **a**–**c**, respectively present the enrichment results for three groups (M versus F1♂, F versus F1♀, P versus F1) of GO terms (including CC, BP, and MF) and KEGG pathways. The vertical axis displays the top 15 significantly enriched entries and pathways, while the horizontal axis represents gene counts. Yellow denotes BP, blue denotes CC, red denotes MF, and green denotes KEGG pathways

This study performed GO functional and KEGG pathway enrichment analyses on DEGs to elucidate their potential roles in biological processes. A comprehensive analysis of the GO enrichment characteristics of the DEGs in each of the three groups (P versus F1, M versus F1♂, and F versus F1♀) was initially performed, revealing their extensive involvement in fundamental aspects of CC, MF, and the regulation of BP. Among the three groups, the most significantly enriched GO terms are primarily distributed across the following categories: in BP, immune response, signal transduction, positive regulation of NF-kappa B transcription factor activity, cellular response to interferon-beta, and defense response to protozoan exhibit significant enrichment; in CC, enrichment is observed in cell surface, membrane, membrane raft, and Golgi apparatus; and in MF, identical protein binding and GTP binding show relatively high enrichment levels (Fig. 1a–c). Notably, upregulated DEGs are associated with distinct GO terms such as membrane and membrane raft (*Arc*), whereas downregulated genes are linked to GO terms including immune response, inflammatory response, Golgi apparatus, and identical protein binding. Genes such as *C4b*, *Cybb*, and *Hvcn1* are consistently located within key enriched functional categories.

**Fig. 2:**
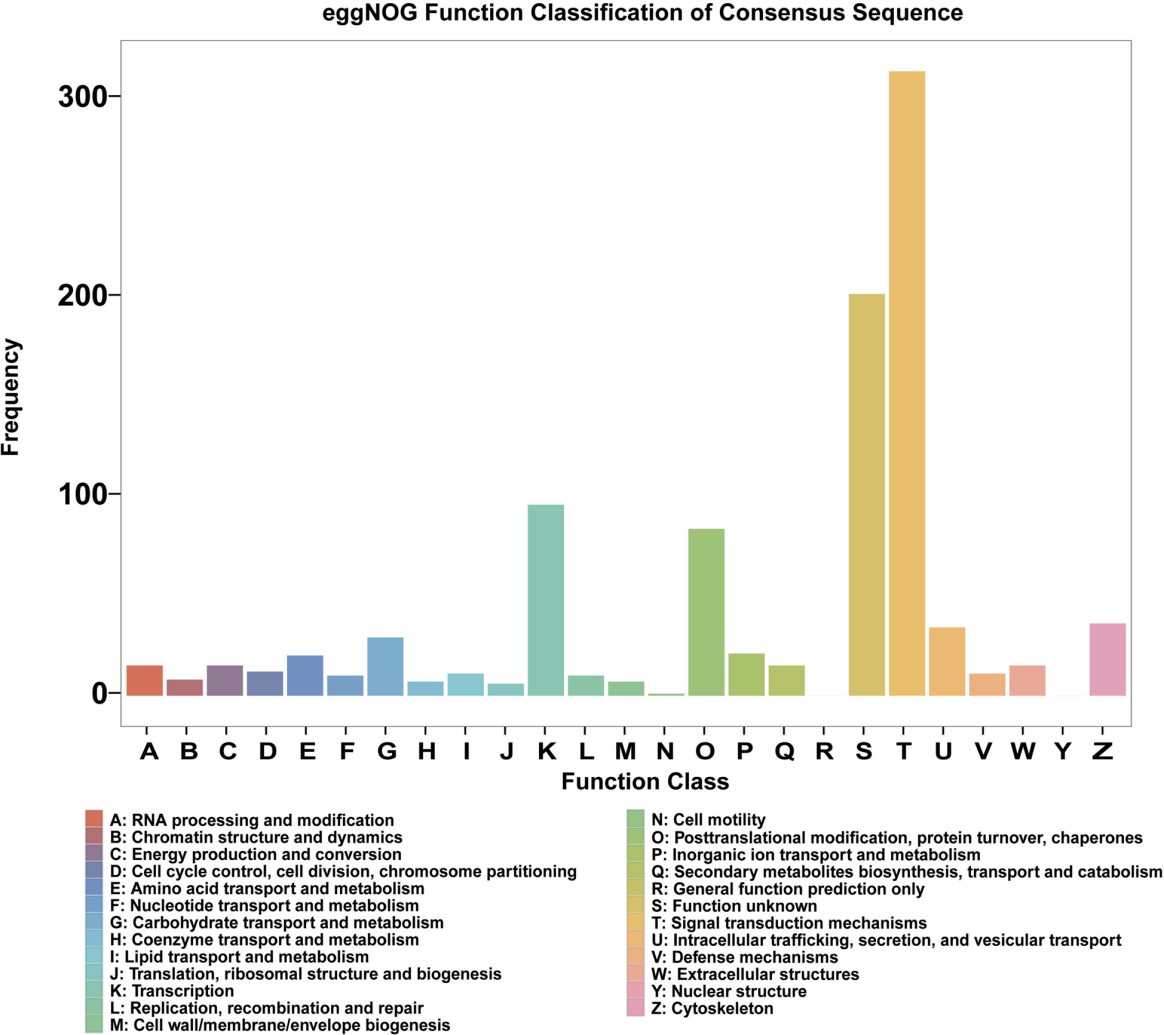
Analysis of eggNOG enrichment of the differentially expressed transcriptome in the brain tissues of chronically *T. gondii*-infected parental mice and their offspring

The KEGG enrichment results are presented in Fig. 2a–c. DEGs are significantly enriched in multiple pathways, with the top 15 including Epstein–Barr virus infection, NOD-like receptor signaling pathway, and toxoplasmosis, all of which are downregulated. Pathways such as osteoclast differentiation, Kaposi’s sarcoma-associated herpesvirus infection, and human T-cell leukemia virus type 1 infection are regulated by both upregulated and downregulated genes. The key gene *Cybb* is enriched in the phagosome pathway, whereas *Itgal* is associated with Epstein–Barr virus infection (Fig. 1a–c).

Furthermore, a total of 24 downregulated genes were identified in the toxoplasmosis-related pathway, primarily including immune-related members of the GTPase family (e.g., *Irgm1*, *Irgm2*, *Igtp*), components of the JAK-STAT signaling pathway (e.g., *STAT1*, *STAT3*), Toll-like receptors (e.g., *Tlr2*, *Tlr4*), and MHC class II antigen-presenting molecules (e.g., *H2-Aa*, *H2-Ab1*), which may collectively influence the immunomodulatory potential of the offspring mice. *Irgm1*, *Irgm2*, and *Igtp* are associated with inflammatory immunity [19, 20], while STAT genes, as downstream effectors of interferon signaling, regulate immune responses via the JAK–STAT pathway [21]. Furthermore, MHC class II molecules exhibited reduced expression following *T. gondii* infection [22]; however, the underlying mechanism of this downregulation in offspring remains to be elucidated. These findings offer novel insights into the impact of *T. gondii* infection on gene expression in host offspring. It is worth noting that due to the complexity of infection, certain GO terms and KEGG pathways did not achieve statistical significance in the F versus F1♀ comparison. This phenomenon also underscores the limitations inherent in the current study, highlighting the need for further validation and expansion in subsequent work.

### Enrichment analysis of eggNOG

A total of 971 DEGs were identified in the eggNOG enrichment analysis of P versus F1, including 109 upregulated and 862 downregulated genes. Among the upregulated genes, the top five most significantly expressed genes were *Nr4a1*, *Egr2*, *Arc*, *Nr4a3*, and *Fos*, which were primarily classified into category K (transcription), with the exception of *Arc*, which belonged to category Z (cytoskeleton). The most significantly downregulated gene was *Cirbp*, predominantly assigned to category T (signal transduction mechanisms). *Cirbp* was classified under category A (RNA processing and modification). Functional classification mapping revealed that category T exhibited the highest gene clustering frequency, followed by category S (function unknown), K, and O (posttranslational modification, protein turnover, chaperones) (Fig. 2). These findings highlight the potential involvement of DEGs in key biological processes such as signal transduction, transcriptional regulation, and RNA processing and modification.

### GSEA analysis

**Fig. 3:**
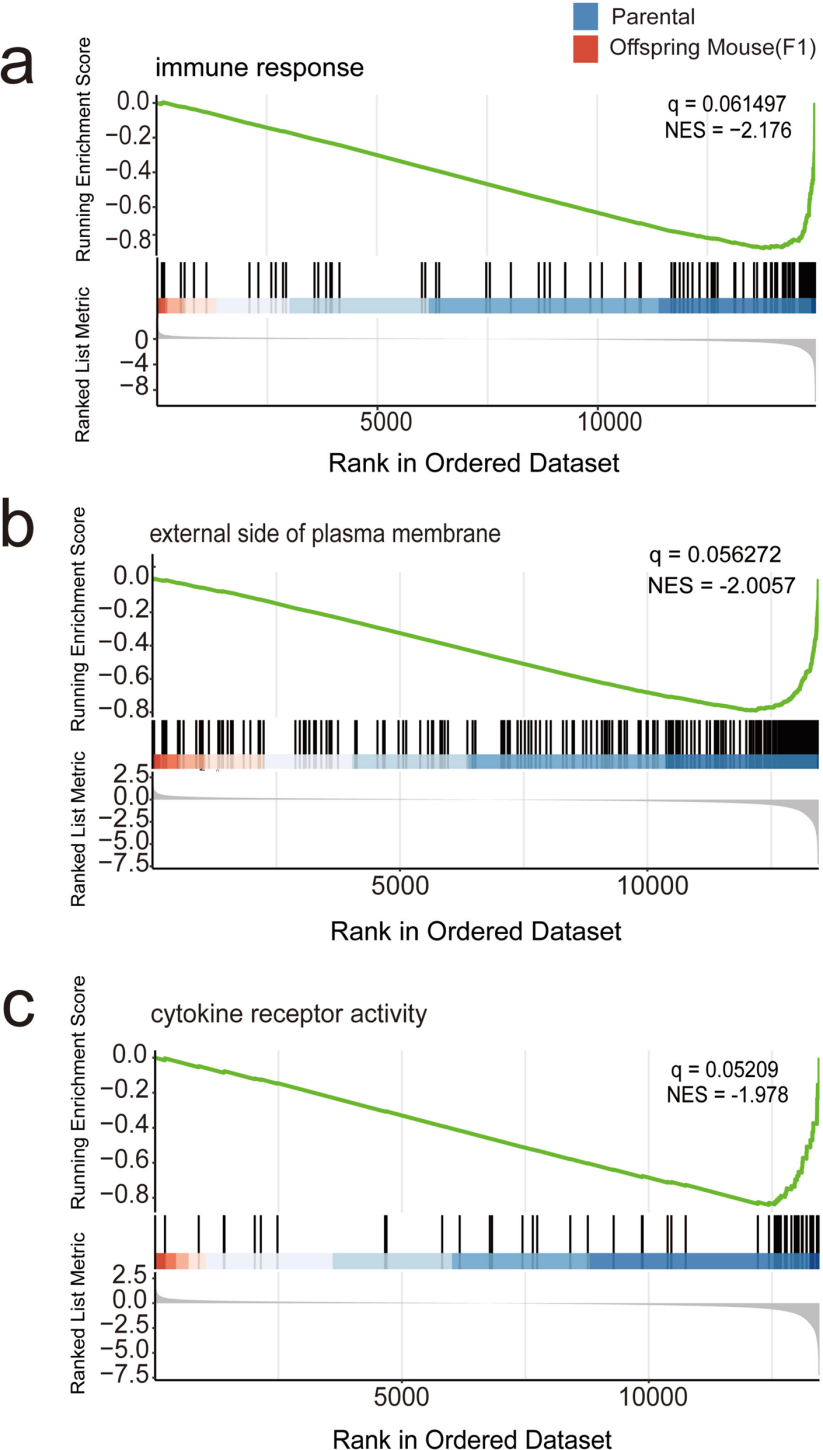
GSEA analysis of the most significant term, with the experimental group depicted in red on the left and the control group illustrated in blue on the right. Shown from top to bottom are the enriched pathways associated with BP (**a**), CC (**b**), and MF (**c**) in the GO enrichment analysis

Using gene sets from the above GO and KEGG pathways, we conducted gene set enrichment analysis (GSEA) for P versus F1. In the GO-BP category, the control gene set showed significant enrichment, with immune response being the most prominent term (|NS|> 2, *Q* < 0.25) (Fig. 3a). In the CC classification, the control gene set was notably enriched in the term “external side of plasma membrane” (|NS|> 2, *Q* < 0.25) (Fig. 3b). In the MF category, the control gene set exhibited significant enrichment, particularly in cytokine receptor activity (|NS|> 1.97, *Q* < 0.25) (Fig. 3c). While immune response was identified as the most significant pathway associated with downregulated biological processes in the previous GO enrichment analysis, cytokine receptor activity showed comparable significance within the molecular function domain.

In the KEGG pathway analysis, several pathways were identified as the most significantly enriched based on the NSE score (i.e., significance), including “I infection”, “Phagosome”, “NOD-like receptor signaling pathway” and “Viral myocarditis” (|NS|> 2, *Q* < 0.25). These findings suggest that the associated gene sets are involved in key biological processes such as immune response, cell membrane function, and viral infection, potentially offering valuable functional insights for future research.

### Verification of the key DEG candidates

**Fig. 4:**
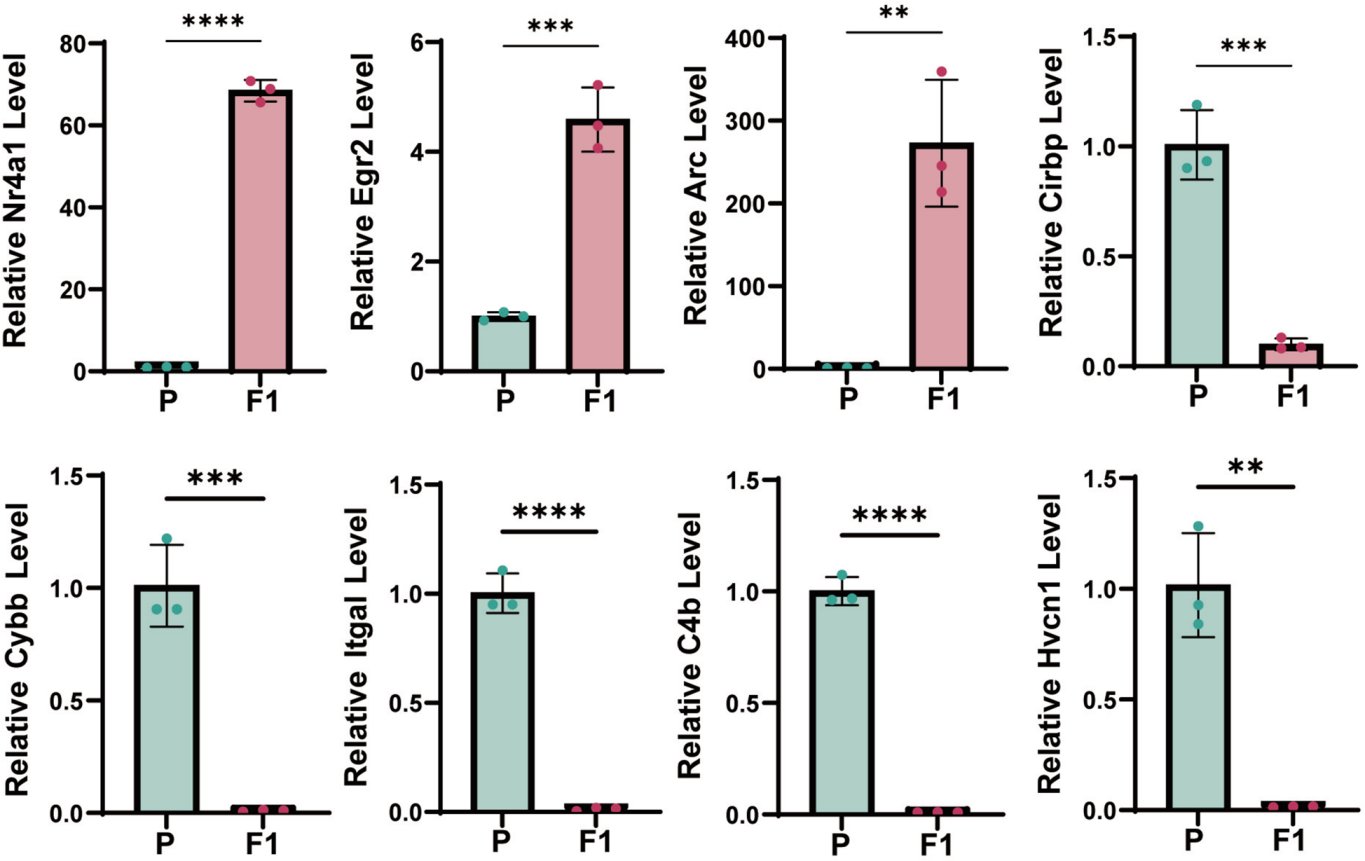
qRT-PCR validation results of significant genes. This study compared brain tissue samples from parental mice (control group; Parental generation) with those from their first-generation offspring (experimental group; F1 generation) (*n *= 4 versus *n* = 3; all biologically independent samples). Data are presented as mean ± standard deviation. Intergroup differences were evaluated using unpaired, two-tailed Student’s *t*-tests; with *P < 0.05 considered statistically significant

To verify the reliability of the overlapping DEGs identified by RNA-seq in all three pairwise comparisons (P versus F1, M versus F1♂, and F versus F1♀), a subset of key genes were validated using qRT-PCR. The validation results demonstrated that the expression changes trends of the majority of the tested genes were highly consistent with those observed in the RNA-seq data. Specifically, the *Nr4a1*, *Arc*, and *Egr2* genes exhibited significantly upregulated expression in both RNA-seq and qRT-PCR assays, while the downregulation of *Cirbp*, *Itgal*, *Cybb*, *Hvcn1*, and *C4b* were also confirmed by qRT-PCR (Fig. 4).

## Discussion

This study aims to demonstrate the transgenerational transmission of specific behavioral phenotypes in the paternal host following *T. gondii* infection. To investigate the underlying molecular mechanisms, RNA-seq analysis was performed on all brain tissue samples. The samples were categorized into three groups (M versus F1♂, F versus F1♀, P versus F1) to exclude the potential sex-related confounding effects. The analysis revealed consistent trends in gene expression changes across all three groups. With respect to pathway enrichment, significant pathways in the M versus F1♂ and P versus F1 groups showed substantial overlap, while fewer significantly enriched pathways were detected in the F versus F1♀ group. Among the 66 DEGs identified as overlapping across all three comparisons, 47 were downregulated and 19 were upregulated. Further, functional enrichment analyses demonstrated that paternal infection may induce widespread downregulation in the transcriptome of offspring brains, particularly affecting immune-related genes. Coincidentally, we have previously demonstrated that the RNA N6-methyladenosine (m^6^A) methylation in the brain tissue of male mice is significantly enhanced following *T. gondii* infection [23]. Meanwhile, N6-methyladenosine, the most prevalent internal mRNA modification in eukaryotes, has been shown to broadly suppress gene expression by promoting mRNA degradation or inhibiting translation under elevated conditions [24, 25]. Therefore, we speculate that the widespread downregulation of gene expression observed in the present study may be associated with a global increase in RNA m^6^A methylation; however, the underlying molecular mechanisms require further investigation.

Among the significantly downregulated genes, those associated with immune function are predominant. The observed downregulation of genes including *Cirbp* (involved in protection against neural damage) [29, 30], *C4b* (associated with immune evasion) [31], *Itgal* (critical for immune synapse formation) [33], *Cybb* (a key regulator of ROS production) [27, 28], and *Hvcn1* (a voltage-gated proton channel) [26] suggest that this inhibitory state may not only impair multiple aspects of immune function and compromise the neuroprotective role of *Cirbp*, but may also result in functional disturbances related to *Hvcn1* activity. This suppressed state may not only impair multiple immune cascade reactions but also disrupt neuroimmune homeostasis, thereby increasing the risk of neuropsychiatric disorders. Specifically, in studies investigating the association between Alzheimer’s disease (AD) and immune mechanisms, multiple key genes have been shown to play functionally interconnected roles. Among these, *Hvcn1* (Hv1 proton channel) is upregulated in activated microglia in AD mouse models. Dysregulation of its expression may contribute to mitochondrial dysfunction and the pathological accumulation of tau protein, potentially underlying the behavioral abnormalities observed in the offspring in this study [26]. Similarly, *Cybb* (NOX2 catalytic subunit), a critical component of reactive oxygen species production in immune cells, has been closely linked to neurodegenerative processes [27, 28]. With respect to immune and infectious mechanisms, *Cirbp* encodes a cold-inducible RNA-binding protein that modulates mRNA stability and translation under stress conditions. Previous studies have reported that overexpression of *Cirbp* confers protection against neural damage in certain stress-induced contexts [29, 30]. *C4b* is not only a key molecule involved in mediating inflammation and synaptic degeneration [31], but also contributes to the phagocytic process that enhances microglial clearance of cellular debris. Furthermore, *T. gondii* can evade host immune responses by binding to C4b-binding protein (C4BP) [32]. Concurrently, *Itgal* encoded integrin LFA-1 (*CD11a*) is highly expressed in peripheral immune cells and plays a critical role in the formation and maintenance of immune synapses [33]. Moreover, *Gpr84* is significantly upregulated in myeloid immune cells during inflammatory conditions and participates in both immunoregulatory and metabolic processes [34]. Collectively, these genes form a crucial molecular network underlying neuroimmunoregulation and disease progression. However, although the roles of those downregulated immune-related key DEGs highlight the intersection of immune function and neural pathophysiology, direct validation within our experimental model remains to be established.

It is noteworthy that the key upregulated genes are predominantly involved in transcriptional regulation and neuronal activity. Therefore, we conducted a systematic analysis to investigate the functional roles of these key upregulated genes and their potential associations with behavioral phenotypes: (ⅰ) *Nr4a1* and *Egr2* are specifically enriched in the functional category of DNA-binding transcription activator activity. *Nr4a3*, a member of the same nuclear receptor family as *Nr4a1*, is also implicated in immune regulation. *Nr4a1* is constitutively expressed at high levels in tolerant T cells, and its overexpression inhibits effector T cell differentiation, whereas its deletion enhances effector function and immune responses by disrupting T cell tolerance. The binding of *Nr4a1* to AP-1 promotes lysine acetylation at position 27 of histone H3 (H3K27ac), thereby activating tolerance-related genes [35]. Further, as an early stress-response factor, *Nr4a3* participates in diverse biological processes, including cellular differentiation and apoptosis, and has been shown to play a critical role in CNS disorders [36]. (ⅱ) *Egr2* is a significantly upregulated gene that promotes TH17 cell differentiation and myeloid cell recruitment in the CNS by enhancing myelomonocytic chemokine expression. In contrast, T cell-specific deletion of *Egr2* attenuates neuroinflammation during host resistance to infection [37]. Moreover, *Egr2* has been associated with age-dependent cognitive impairment and hereditary neuropathies [38, 39]. (ⅲ) *Arc* is an activity-dependent gene whose expression is closely linked to neuronal excitability and significant behavioral activity. *Arc* knockout mice exhibit impaired long-term memory across multiple behavioral paradigms, while short-term memory remains intact, indicating a critical role for *Arc* in the consolidation of long-term memory [40]. Moreover, *Arc* contributes significantly to cognitive functions, potentially through selective modulation of glutamate and dopamine signaling pathways [41]. Caroline et al*.* demonstrated that *Arc* forms a complex with βSpIV∑5 and, upon binding to PML nuclear bodies—an epigenetic regulatory site—enhances histone acetylation mediated by Tip60 at H4K12. These findings support an epigenetic mechanism by which *Arc* regulates memory consolidation [42]. (ⅳ) *Fos* encodes a leucine zipper protein that contributes to the formation of hippocampal place codes and contextual memory traces by generating precise, stable, and spatially consistent neural representations in the hippocampus [43]. The expression patterns of these key upregulated genes provide molecular insights into the behavioral phenotypes observed in the offspring. As pivotal regulators of both the immune and nervous systems, the upregulation of *Nr4a1/3* and *Egr2* may alter the baseline neural state in offspring through remodeling of the immune–brain axis function. Concurrently, altered expression of *Arc* and *Fos*—core genes involved in synaptic plasticity and neuronal activation—is directly associated with the observed deficits in spatial learning and memory. These changes may reflect compensatory adaptations or subtle imbalances in neural circuit function within the context of transgenerational inheritance.

Functional enrichment analysis further indicates that pathways associated with neuronal activity and stress responses are significantly activated, primarily including DNA-binding transcription factor activity (e.g., *Nr4a1*, *Egr2*) and immune response pathways. Concurrently, eggNOG functional classification results summarize this overall trend: enhanced transcriptional regulatory activity accompanied by reduced immune and signal transduction functions. Based on these findings, we propose a “metabolic reprogramming” hypothesis: parental *T. gondii* infection induces epigenetic reprogramming in germ cells, thereby leading to reprogramming of the offspring brain transcriptional program, which manifested as a predominantly suppressive gene expression pattern. The reprogramming of the offspring brain transcriptome may negatively regulate the neuroimmune system and neurological functions, and induce behavioral abnormalities in the offspring similar to those observed in the parents. Future studies involving direct profiling of epigenetic modifications in sperm from infected males and in the brains of their offspring will be essential for validating this hypothesis.

Therefore, this study provides preliminary evidence that contributes to the understanding of the transgenerational immune and neuroregulatory effects of *T. gondii* infection. However, given that severe reproductive dysfunction in males has been documented following chronic *Toxoplasma* infection, all offspring analyzed in this study were derived from a single infected male. Thus, although our findings demonstrate internal consistency and offer valuable insights within this specific pedigree, they should be interpreted with caution and may not be fully generalizable to broader populations or diverse genetic backgrounds. Furthermore, to assess the potential influence of sex on offspring behavior, we performed the open field test (evaluating anxiety-like behavior) and the Morris water maze test (evaluating spatial learning and memory) in advance on the offspring mice (Additional File 3: Supplementary Fig. S1). The results demonstrated no statistically significant sex-dependent differences in the measured behavioral parameters. This further confirms the major transcriptional signature identified, the widespread downregulation of specific immune and signaling pathways in offspring, is meaningfully linked to paternal *T. gondii* infection. Future studies should include offspring sired by multiple infected males to confirm and extend these observations.

## Conclusions

We found that *T. gondii* infection of male parental mice significantly downregulates gene expression in the brain tissue of their offspring and negatively regulates the immune system, developmental processes, and neurological functions. Among key DEGs, *Egr2*, *Arc*, and *Fos* are closely associated with neuropsychiatric disorders. And *Cirbp*, *Cybb*, *Itagl*, *Hvcn1*, and *Gpr84* were identified as key regulators of immune response and may also exert potential influences on psychiatric conditions. Although the precise mechanisms underlying this phenomenon in humans remain to be elucidated, we believe these findings highlight the potential long-term impact of paternal infection on offspring health and offer meaningful insights for guiding future research.

## Supplementary Information

Additional file1: Table S1. Quality control results analysis of the sample

Additional file2: Table S2. Alignment of sample brain transcriptome sequencing data with reference genomes

Additional file3: Figure S1. The behavioral studies showed that both the male and female offspring mice exhibited similar behavioral changes. **a** The open field experiment results indicated that there was no difference in the number of times and duration of staying in the central area between the male and female offspring mice, suggesting that both the male and female offspring mice were in an anxious state. **b** The Y-maze results showed that there was no statistical difference in the total number of arm entries and spontaneous conversion rates between the male and female offspring mice, indicating that the degree of spatial memory impairment in the male and female offspring mice was similar. **c** The water maze results showed that there was no difference in the number of times crossing the platform and the escape latency between the male and female offspring mice, meaning that the male and female offspring mice exhibited similar spatial learning and memory impairments.

## Data Availability

The datasets supporting the findings of this article are included within the paper and its supplementary materials.

## Abbreviations

*T. gondii*: *Toxoplasma gondii*
*TgCtwh6*: Chinese cat-derived type Ⅰ *TgCtwh6* Strain of *T. gondii*
DEGs: Differentially expressed genes
GO: Gene ontology
BP: Biological process
CC: Cellular component
MF: Molecular function
KEGG: Kyoto encyclopedia of genes and genomes
EggNOG: Evolutionary genealogy of genes: non-supervised orthologous groups
qRT-PCR: Quantitative reverse transcription polymerase chain reaction
Nr4a1: Nuclear receptor subfamily 4 group a member
Nr4a3: Nuclear receptor subfamily 4 group a member3
Egr2: Early growth response protein 2
Arc: Activity-regulated cytoskeleton-associated protein
Fos: Fos proto-oncogene, AP-1 transcription factor subunit
Cirbp: Cold-inducible RNA binding protein
Itgal: LFA-1, Lymphocyte function-associated antigen 1
Cybb: Cytochrome b-245 heavy chain
Hvcn1: Hydrogen voltage gated channel 1
Gpr84: G protein-coupled receptor 84
GSEA: Gene set enrichment analysis
AD: Alzheimer’s disease
H3K27ac: Lysine acetylation at position 27 of histone 3
CNS: Central nervous system
