# Supplementary material for: The effect of Toxoplasma gondii infection in parental male mice on the transcriptome of their offspring’s brain
Source: Parasit Vectors. 2026 Feb 26;19:142. doi: 10.1186/s13071-026-07302-7 (PMC13040779; doi:10.1186/s13071-026-07302-7)
Supplement: Supplementary file 1 — Additional file1: Table S1. Quality control results analysis of the sample [file 13071_2026_7302_MOESM1_ESM.docx]

Supplement Information

Table 1: Quality control results analysis of the sample

| Sample ID | Read Sum | Base Sum | GC(%) | N(%) | Q20(%) | Q30(%) |
| --- | --- | --- | --- | --- | --- | --- |
| A1 | 25456993 | 7611658016 | 48.57 | 0.04 | 98.94 | 95.87 |
| A2 | 23956512 | 7153665728 | 48.74 | 0.05 | 98.89 | 95.79 |
| A3 | 21703971 | 6482547124 | 48.77 | 0.03 | 99.15 | 96.71 |
| B1 | 23354632 | 6982141896 | 48.63 | 0.05 | 98.85 | 95.54 |
| B2 | 21478115 | 6416312464 | 48.56 | 0.05 | 98.71 | 95.17 |
| B3 | 23075373 | 6900465870 | 48.37 | 0.05 | 99.08 | 96.48 |
| F1 | 21347058 | 6381517314 | 49.01 | 0.05 | 99.06 | 96.44 |
| M1 | 21576206 | 6453493472 | 48.29 | 0.05 | 98.85 | 95.64 |
| M2 | 20680713 | 6182528048 | 48.52 | 0.05 | 98.87 | 95.74 |
| M3 | 26441899 | 7902234144 | 48.62 | 0.04 | 98.96 | 95.93 |
