# Supplementary material for: The effect of Toxoplasma gondii infection in parental male mice on the transcriptome of their offspring’s brain
Source: Parasit Vectors. 2026 Feb 26;19:142. doi: 10.1186/s13071-026-07302-7 (PMC13040779; doi:10.1186/s13071-026-07302-7)
Supplement: Supplementary file 2 — Additional file2: Table S2. Alignment of sample brain transcriptome sequencing data with reference genomes [file 13071_2026_7302_MOESM2_ESM.docx]

Supplement Information

Table 2: Alignment of sample brain transcriptome sequencing data with reference genomes

| Sample | Total Reads | Mapped Reads | Uniq Mapped Reads | Multiple Map Reads | Reads Map to “+” | Reads Map to “-” |
| --- | --- | --- | --- | --- | --- | --- |
| A1 | 50,913,986 | 49,329,866  (96.89%) | 46,997,325  (92.31%) | 2,332,541  (4.58%) | 25,973,780  (51.02%) | 26,008,436  (51.08%) |
| A2 | 47,913,024 | 46,355,266  (96.75%) | 43,806,438  (91.43%) | 2,548,828  (5.32%) | 24,588,964  (51.32%) | 24,637,635  (51.08%) |
| A3 | 43,407,942 | 42,214,563  (97.25%) | 39,711,403  (91.48%) | 2,503,160  (5.77%) | 22,491,085  (51.81%) | 22,510,121  (51.86%) |
| B1 | 46,709,264 | 45,213,833  (96.80%) | 42,719,815  (91.46%) | 2,494,018  (5.34%) | 23,987,836  (51.36%) | 24,014,922  (51.41%) |
| B2 | 42,956,230 | 41,353,555  (96.27%) | 38,948,539  (90.67%) | 2,405,016  (5.60%) | 21,983,092  (51.18%) | 22,028,943  (51.28%) |
| B3 | 46,150,746 | 44,699,849  (96.86%) | 42,057,833  (91.13%) | 2,642,016  (5.72%) | 23,798,421  (51.57%) | 23,819,494  (51.61%) |
| F1 | 42,694,116 | 41,312,552  (96.76%) | 39,160,896  (91.72%) | 2,151,656  (5.04%) | 21,874,415  (51.24%) | 21,915,087  (51.33%) |
| M1 | 43,152,412 | 41,749,121  (96.75%) | 39,142,794  (90.71%) | 2,606,327  (6.04%) | 22,287,247  (51.65%) | 22,320,122  (51.72%) |
| M2 | 41,161,426 | 39,986,996  (96.68%) | 37,505,184  (90.68%) | 2,481,812  (6.00%) | 21,349,458  (51.62%) | 21,384,721  (51.70%) |
| M3 | 52,883,798 | 51,303,720  (97.01%) | 47,972,311  (90.71%) | 3,331,409  (6.30%) | 27,481,366  (51.97%) | 27,505,282  (52.01%) |

Note: (Mouse reference genome: GRCm38 (MusMusculus)) A1~3: offspring male mice 1~3; B1~3: offspring female mice; F1: parental infected male mice; M1~3: parental normal female mice.
