# Supplementary figures and images for: The effect of Toxoplasma gondii infection in parental male mice on the transcriptome of their offspring’s brain
Source: Parasit Vectors. 2026 Feb 26;19:142. doi: 10.1186/s13071-026-07302-7 (PMC13040779; doi:10.1186/s13071-026-07302-7)

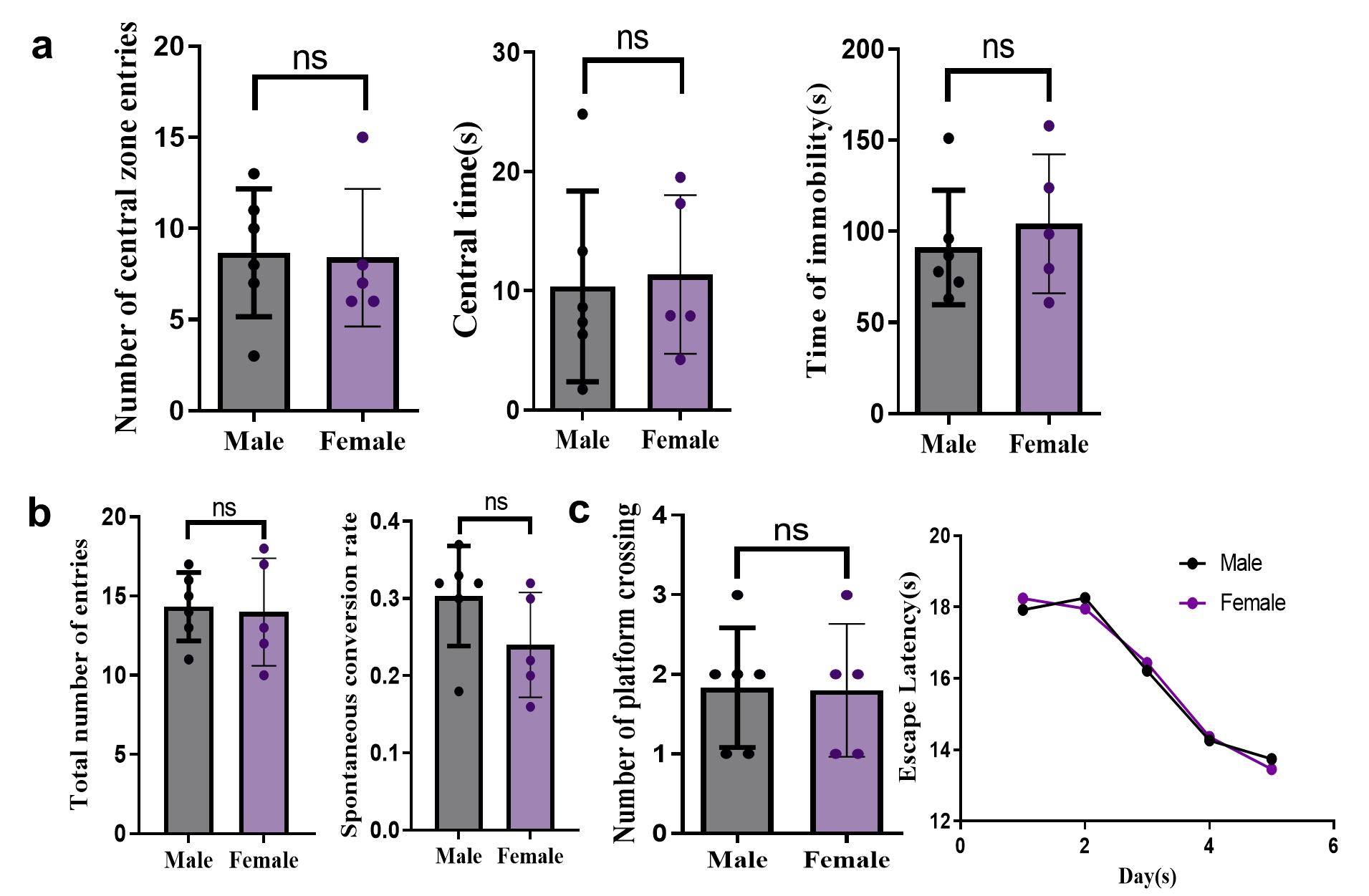

Supplement: Supplementary file 3 — Additional file3: Figure S1. The behavioral studies showed that both the male and female offspring mice exhibited similar behavioral changes. a The open field experiment results indicated that there was no difference in the number of times and duration of staying in the central area between the male and female offspring mice, suggesting that both the male and female offspring mice were in an anxious state. b The Y-maze results showed that there was no statistical difference in the total number of arm entries and spontaneous conversion rates between the male and female offspring mice, indicating that the degree of spatial memory impairment in the male and female offspring mice was similar. c The water maze results showed that there was no difference in the number of times crossing the platform and the escape latency between the male and female offspring mice, meaning that the male and female offspring mice exhibited similar spatial learning and memory impairments. [file 13071_2026_7302_MOESM3_ESM.tif]
